# Development of a Biodegradable PLGA Carrier to Provide Wnt Agonists and Antibiotics to Meet the Requirements for Patients with Bone Infections

**DOI:** 10.3390/ph17081038

**Published:** 2024-08-06

**Authors:** Song-Shu Lin, Shih-Jung Liu, Err-Cheng Chan, Kowit-Yu Chong, Yi-Sheng Chan, Tsung-Ting Tsai, Chi-Chien Niu, Li-Jen Yuan, Chuen-Yung Yang, Hui-Yi Hsiao, Yi-Jen Hsueh, Chung-An Chen, Steve W. N. Ueng

**Affiliations:** 1 Department of Orthopaedic Surgery, Chang Gung Memorial Hospital, Taoyuan 333, Taiwantsai1129@gmail.com (T.-T.T.);; 2 Department of Nursing, Chang Gung University of Science and Technology, Taoyuan 333, Taiwan; 3 Hyperbaric Oxygen Research Lab, Bone and Joint Research Center, Chang Gung Memorial Hospital, Taoyuan 333, Taiwan; kchong@mail.cgu.edu.tw; 4 Department of Mechanical Engineering, Chang Gung University, Taoyuan 333, Taiwan; shihjung@mail.cgu.edu.tw; 5 Department of Medical Biotechnology and Laboratory Science, Chang Gung University, Taoyuan 333, Taiwan; 6 College of Medicine, Chang Gung University, Taoyuan 333, Taiwan; 7 Department of Orthopaedic Surgery, E-Da Hospital, I-Shou University, Kaohsiung 82445, Taiwan; 8 Center for Tissue Engineering, Chang Gung Memorial Hospital, Taoyuan 333, Taiwan; 9 Department of Biomedical Science, Chang Gung University, Taoyuan 333, Taiwan; 10 Department of Ophthalmology, Chang Gung Memorial Hospital, Taoyuan 333, Taiwan

**Keywords:** polylactide–polyglycolide, vancomycin, lithium, drug delivery, WNT agonists, mesenchymal stem cells

## Abstract

Antibiotic beads can be used to treat surgical infections. In this study, polylactide–polyglycolide (PLGA) was mixed with vancomycin, the osteogenic enhancer lithium chloride (LiCl), and hot compression to form PLGA-vancomycin-LiCl delivery beads to treat bone infection. An elution method was used to characterize in vitro release characteristics of vancomycin and Li over a 42-day period. The release profiles lasted for more than 42 days for vancomycin and 28 days for Li. The concentration of vancomycin in each sample was well above the breakpoint sensitivity. Lithium cotreatment enhanced the bactericidal effect of vancomycin. Released Li and vancomycin increased the mRNA or protein expressions of osteogenic markers of mesenchymal stem cells (MSCs). In vivo, the PLGA delivery systems were implanted into the distal femoral cavities of rabbits, and the cavity fluid content was aspirated and analyzed at each time point. The released Li and vancomycin lasted more than 6 weeks, and the vancomycin concentrations were much greater than the breakpoint sensitivity. Four rabbits in each group were sacrificed at 8 weeks for histological observation. More mature bone tissue was observed in the Li treatment group. This study provides a PLGA drug delivery system to meet the requirements of patients with bone infections.

## 1. Introduction

Despite advances in surgical techniques and the availability of newly developed antibiotics, bone infections after surgical procedures continue to be a difficult problem for surgeons [1]. Currently, the combination of surgical intervention and an effective antibiotic remains the standard treatment [2]. Systemic and topical antibiotic therapies are usually used [3,4]. The most common of these is the incorporation of antibiotics into polymethylmethacrylate (PMMA) [5,6], an approach that was originally used by Buchholz et al. [2]. The antibiotic-impregnated PMMA beads provide a high concentration of local antibiotics and have advantages over intravenous injection, such as low systemic levels, therefore producing minimal allergic reactions and systemic complications. Antibiotic-impregnated PMMA beads provide a high concentration of topical antibiotics and have the advantage of low systemic levels over intravenous administration, resulting in minimal allergic reactions and systemic complications. However, PMMA beads may require a second operation for removal after prolonged implantation [1,3].

A biodegradable carrier such as a slow-release bead for delivering antibiotics may be better than PMMA beads and intravenous antibiotics. Biodegradable beads provide long-term bactericidal concentrations of antibiotics, and their biodegradability can be altered from weeks to months to treat many types of infections. Since the biodegradable beads dissolve gradually and the soft tissue or bone defect slowly fills with tissue, it is thus unnecessary for bone/tissue reconstruction [7]. A large number of biocompatible and biodegradable substrates, such as bioceramics, polymers, bioglasses, and composite materials, have been tested as possible methods for the use of topical antibiotics to treat osteomyelitis in vitro and in vivo [8]. Poly (lactide-co-glycolide) (PLGA) copolymers are favorable biodegradable materials. PLGA induces a minimal inflammatory response, is nontoxic, and can be absorbed with no accumulation in vital organs. PLGA has been used as a carrier material for antibiotics [9]. In 1999, researchers used a hot compression molding method to fabricate PLGA antibiotic beads and achieved in vitro antibiotic release lasting more than 32 days [8,9]. Additionally, antibiotic release for 56 days from PLGA-vancomycin beads in a rabbit model was reported in 2002 [10]. In 2007, compression sintering and ultrasonic welding techniques were used to produce biodegradable polymer capsules that could simultaneously release vancomycin and rhBMP-2 [11]. In 2016, PLGA antibiotic beads were used to eradicate *Staphylococcus aureus* (*S. aureus*) infection in damaged bone [12].

Lithium ion (Li^+^) is a well-known essential trace element used as a mood stabilizer and found in cereals, vegetables, and drinking water [13,14]. Lithium has been used to treat bipolar disorder, and numerous studies have shown that lithium can prevent apoptosis, prolong lifespan, and is widely used as an anticancer drug in combination therapy [15]. In addition, lithium affects the proliferation of hematopoietic stem cells (HSCs) and its protective effect against cadmium, another toxic metal, has been previously reported [15]. Recent studies have shown that Li increases the proliferation of mesenchymal stem cells (MSCs), stimulates osteogenesis, and inhibits the adipogenic differentiation of MSCs by stimulation of the Wnt/β-catenin pathway [16]. To provide a solution for infected bone defects, Li-modified PLGA antibiotic beads could be used for bone restoration and defect augmentation. The PLGA antibiotic beads used for bone repair are biodegradable, bioactive, and biocompatible. Lithium detoxification is at least partly mediated by Li^+^ efflux via a Na^+^/H^+^ antiporter in *E*. *coli* [17]. Previous studies have shown that the size of the inhibition zone caused by vancomycin can determine the ability of vancomycin to inhibit bacterial growth. However, the combined effects of lithium and vancomycin on the inhibition zone and relative activity of vancomycin against *S. aureus* are not clear. The possible molecular mechanism is worth future exploration.

In this study, we developed PLGA-vancomycin-LiCl delivery beads for the treatment of bone infection. The objective of the present study was (I) to prepare sustained release Li and antibiotic beads using matrix materials including PLGA, LiCl, and vancomycin; (II) to examine the in vitro release characteristics of lithium and vancomycin from formulated beads; (III) to investigate the effects of bead elution on the osteogenesis of MSCs; (IV) to investigate the effects of bead elution on bioassays of antibacterial activity; (V) to examine the in vivo release characteristics of Li and vancomycin from formulated beads in a rabbit model; and (VI) to examine the repaired tissues of the rabbits following bead treatment.

## 2. Results

### 2.1. Fabrication of Biodegradable PLGA-Vancomycin-LiCl Beads

**Figure 1:**
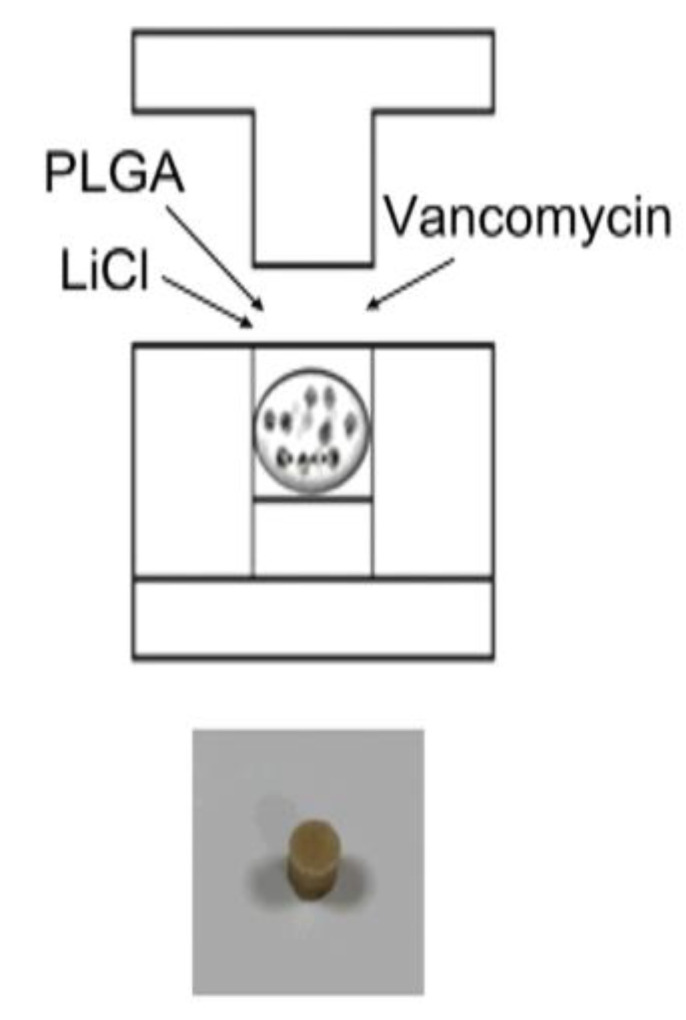
Fabrication of biodegradable PLGA-vancomycin-LiCl beads.

The PLGA, vancomycin, and LiCl mixture was hot-compressed into drug delivery beads 5 mm in diameter using a mold, as shown in Figure 1. Four types of PLGA drug delivery systems were investigated in this study: Type I, PLGA-vancomycin beads without LiCl; Type II, PLGA-vancomycin-10 mg LiCl beads; Type III, PLGA-vancomycin-50 mg LiCl beads; and Type IV, PLGA-vancomycin-100 mg LiCl beads.

### 2.2. In Vitro Release Dynamics of PLGA-Vancomycin-LiCl Beads

**Figure 2:**
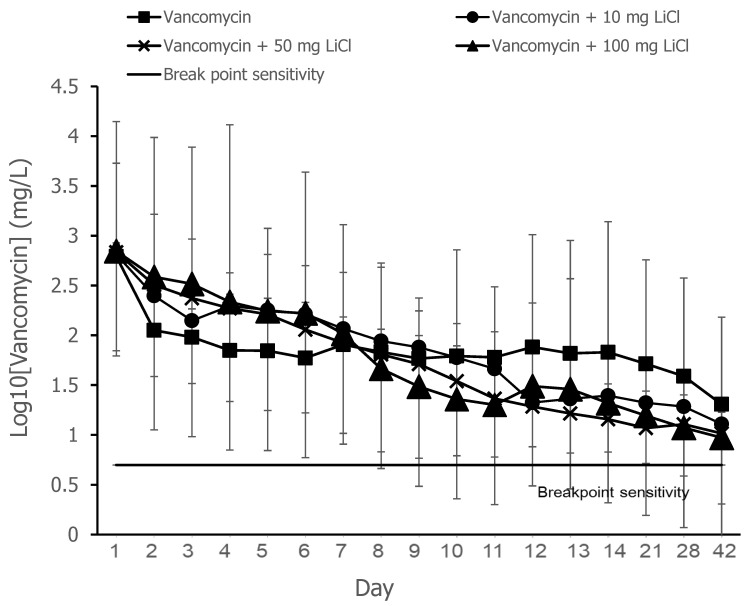
Release curves of vancomycin from the PLGA-vancomycin-LiCl beads. Four types of PLGA drug delivery beads were investigated in this study: Type I, PLGA-vancomycin beads without LiCl (▓); Type II, PLGA-vancomycin-10 mg LiCl beads (●); Type III, PLGA-vancomycin-50 mg LiCl beads (X); Type IV, PLGA-vancomycin-100 mg LiCl beads (▲).

The release curves of vancomycin from the PLGA-vancomycin-LiCl delivery beads are shown in Figure 2. Data from three samples were analyzed for each test. The concentrations of released vancomycin were most evident during the first two days. The breakpoint sensitivity of vancomycin for *S. aureus* was 5 mg/L. The gradual elution of vancomycin from the drug delivery beads was observed throughout the duration and remained above the breakpoint sensitivity level until Day 42.

**Figure 3:**
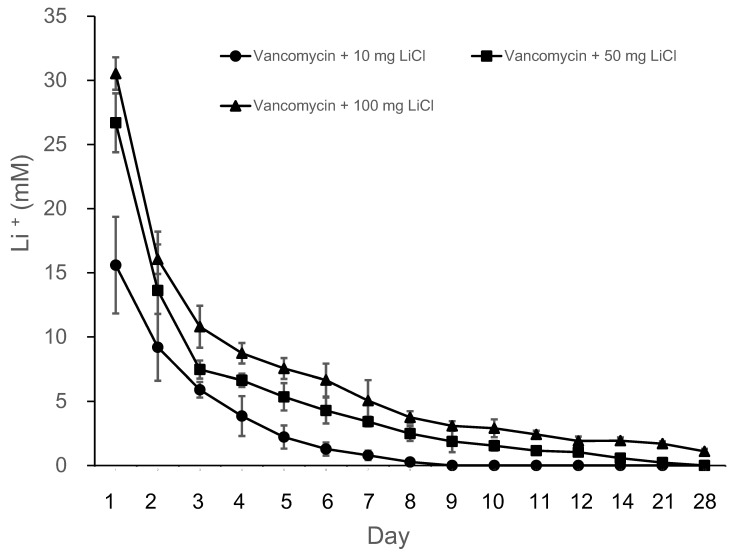
Release curves of lithium from the PLGA-vancomycin-LiCl beads.

The release curves of lithium (Li) from the PLGA-vancomycin-LiCl delivery beads are shown in Figure 3. Data from three samples were analyzed for each test. The mean Li concentrations on Days 1, 2, 4, 7, 10, 14, 21, and 28 from the Type II beads were 15.6, 9.2, 3.5, 0.79, 012, 0, 0, and 0 mM, respectively; those from the Type III beads were 26.7, 13.6, 6.6, 3.4, 1.54, 0.23, 0, and 0 mM, respectively; and those from the Type IV beads were 30.5, 16.1, 8.7, 5.1, 2.9, 1.7, 1.1, and 0.3 mM, respectively. Li was gradually released from the Type II beads for 10 days, Type III beads for 14 days, and Type IV beads for 28 days. Lithium release was the most obvious during the first two days.

### 2.3. Relative Activity Assay of the Collected Eluant from PLGA-Vancomycin-LiCl Beads

**Figure 4:**
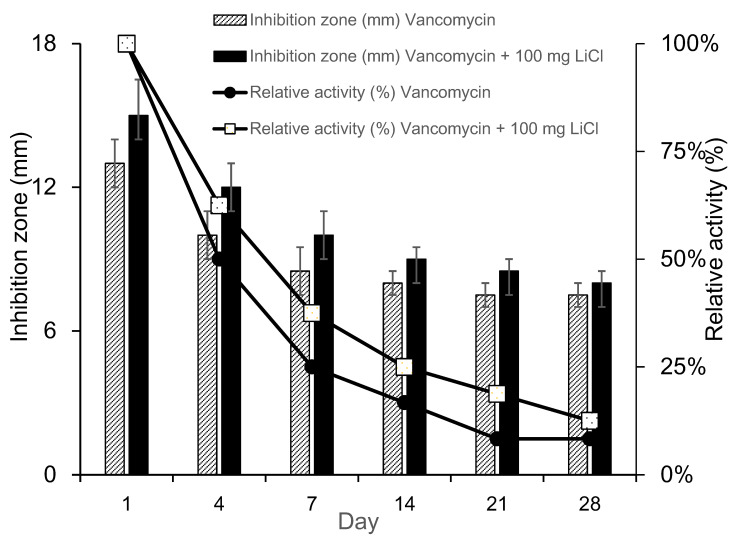
Relative activity of collected elution from PLGA-vancomycin-LiCl beads.

The results of the PLGA-vancomycin delivery beads are shown in Table 1. The sample inhibition zone and relative activity were greater for Type IV beads than for Type I beads at each time point. Compared with that of Type I beads, the bactericidal effect of vancomycin was enhanced by Li cotreatment in Type IV beads (Figure 4). The release of LiCL from the carrier affected the release of antibiotics and increased the relative activity of antibiotics.

### 2.4. Promotion of MSC Osteogenesis via Elution of the PLGA-Vancomycin-LiCl Beads

**Figure 5:**
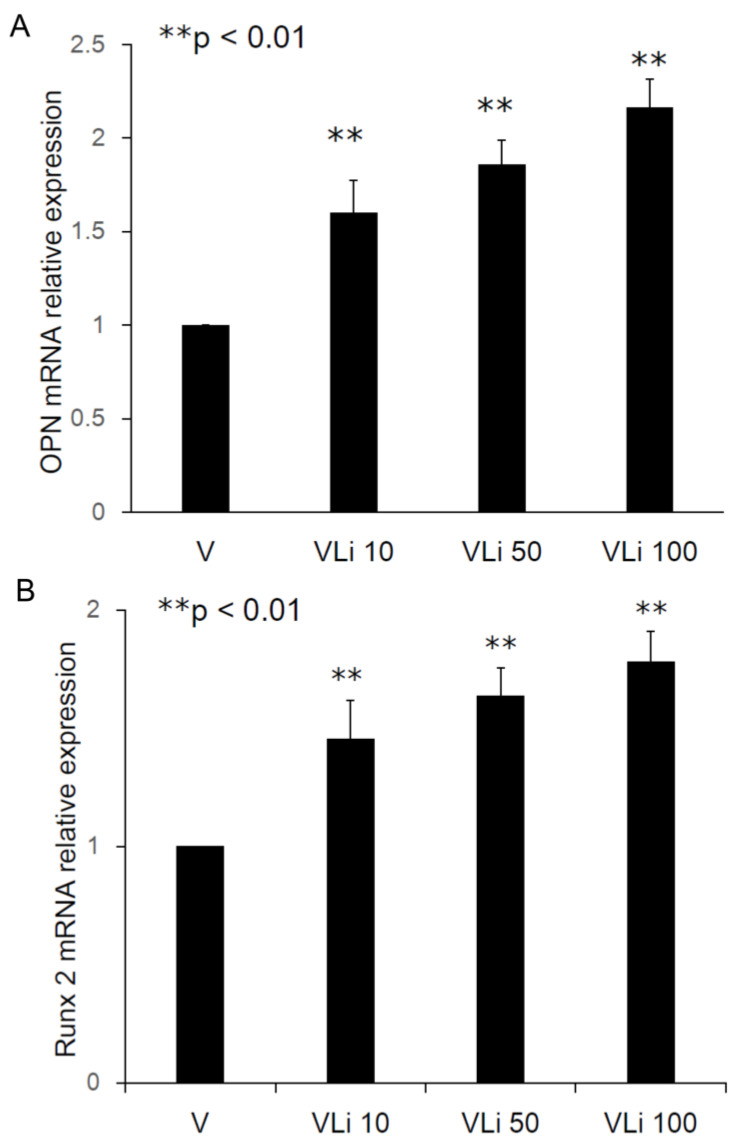
Promotion of osteogenesis of MSCs by increasing in osteopontin (**A**) and Runx2 (**B**) mRNA expression.

**Figure 6:**
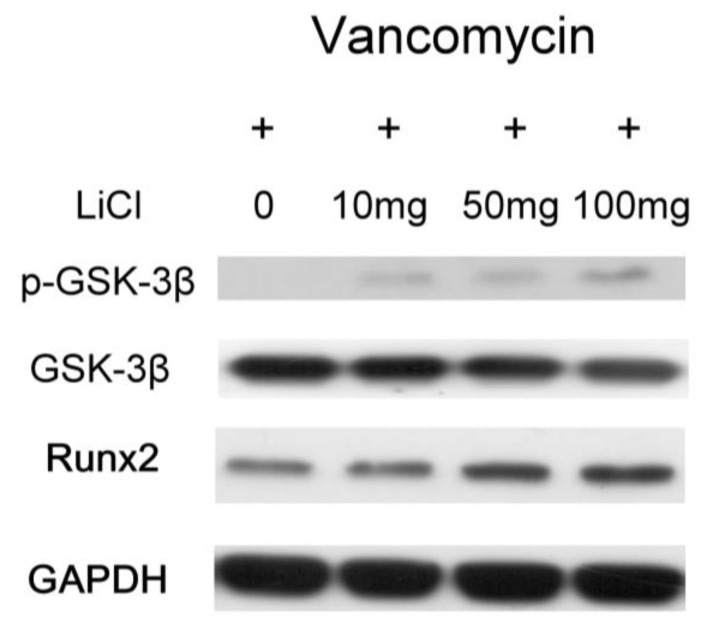
Promotion of osteogenesis of MSCs by increasing in phosphorylation of GSK-3β protein and Runx 2 protein expression.

The relative osteopontin (OPN) mRNA expression ratios were 1.60 ± 0.17 fold (Type II beads/Type I beads, ** *p* < 0.01, n = 3), 1.86 ± 0.13 fold (Type III beads/Type I beads, ** *p* < 0.01, n = 3), and 2.16 ± 0.15 fold (Type IV beads/Type I beads, ** *p* < 0.01, n = 3) (Figure 5A). The relative Runx2 mRNA expression ratios were 1.45 ± 0.16 fold (Type II beads/Type I beads, ** *p* < 0.01, n = 3), 1.64 ± 0.12 fold (Type III beads/Type I beads, ** *p* < 0.01, n = 3), and 1.78 ± 0.13 fold (Type IV beads/Type I beads, ** *p* < 0.01, n = 3) (Figure 5B). After treatment of the MSCs by elution from the PLGA-vancomycin-LiCl beads, the released Li promoted the osteogenesis of the MSCs by increasing the mRNA expression of osteopontin (OPN) (Figure 5A) and Runx2 (Figure 5B). In addition, the released Li was observed to increase the phosphorylation of the GSK-3β protein and upregulate the expression of the Runx 2 protein (Figure 6). These two results indicate that increased Li tends to promote the ossification of MSCs; however, additional observations are still needed to demonstrate this effect.

### 2.5. Animal Model of Bone Defect

**Figure 7:**
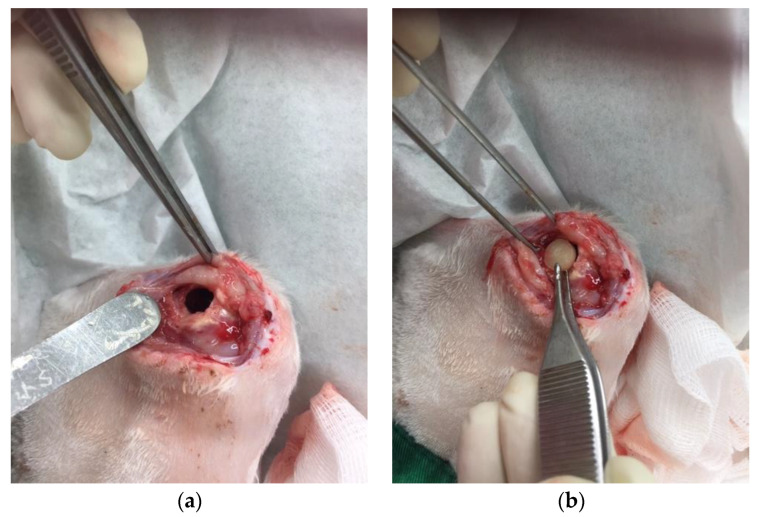
(**a**) A cylindrical cavity was made. (**b**) One PLGA-vancomycin-LiCl bead was inserted into the cavity.

A cylindrical cavity (15 mm × 12 mm × 10 mm) was made at the distal end of the right femur and obliterated with a PMMA spacer. The wound was closed with sutures. After 2 weeks, the PMMA spacer was removed, one composite delivery bead was inserted into the cavity, and the wound was subsequently closed (Figure 7a,b).

#### 2.5.1. In Vivo Elution Assay for Lithium

**Figure 8:**
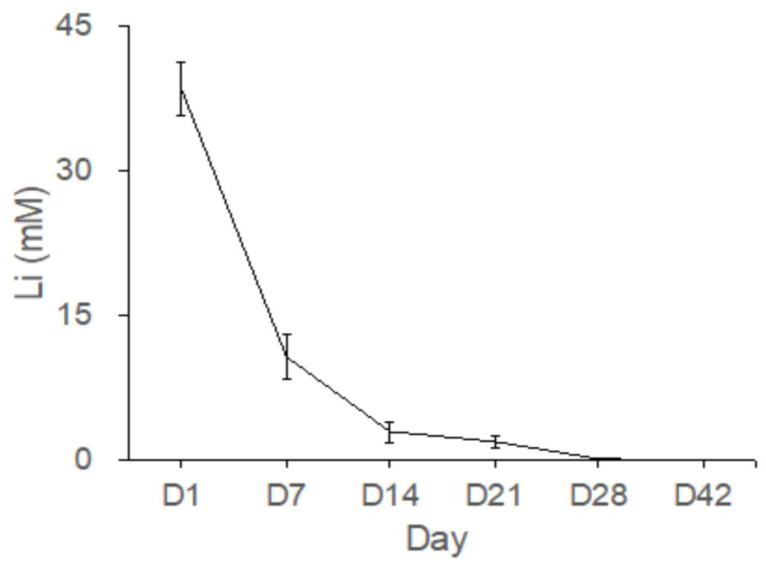
Gradual release of Li from the Type IV beads for more than 42 days in vivo.

Figure 8 shows the Li concentrations as measured in the bone cavity tissue. The mean Li concentrations eluted from the Type IV beads in the femoral cavities on Days 1, 7, 14, 21, 28, and 42 were 38.5, 8.5, 3.7, 1.7, 0.2, and 0.04 mM, respectively (Figure 8). Gradual release of Li was detected from Type IV beads for up to 42 days in vivo. The concentrations of released Li were most evident during the first 24 h.

#### 2.5.2. In Vivo Elution Assay for Vancomycin

**Figure 9:**
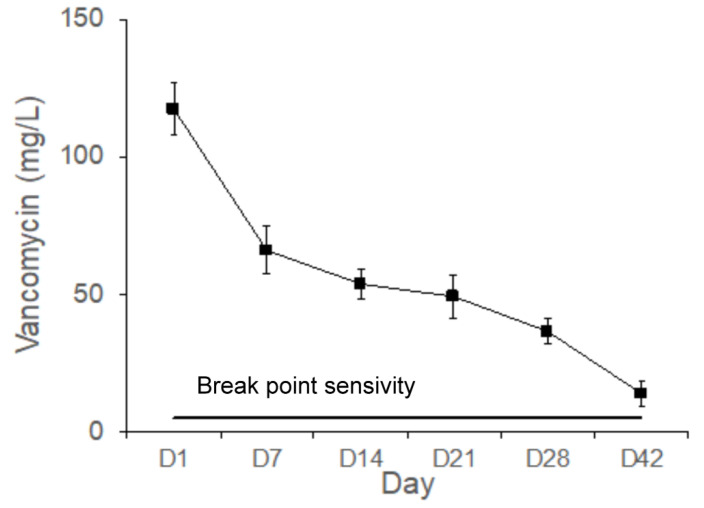
Gradual release of vancomycin from the Type IV beads for up to 42 days in vivo.

Figure 9 shows the vancomycin concentrations measured in the bone cavity tissue. The mean concentrations of vancomycin eluted from the Type IV beads in the femoral cavities on Days 1, 7, 14, 21, 28, and 42 were 117.4, 66.0, 56.2, 49.2, 36.4, and 13.8 mg/L, respectively. The gradual release of vancomycin is shown from Type IV beads and above the breakpoint sensitivity for up to 42 days in vivo. The released vancomycin concentrations exhibited the most marked differences during the first 24 h. The breakpoint sensitivity of vancomycin for *S. aureus* was 5 mg/L. The local concentrations of vancomycin were much higher than the breakpoint sensitivity.

### 2.6. Histologic Observation

**Figure 10:**
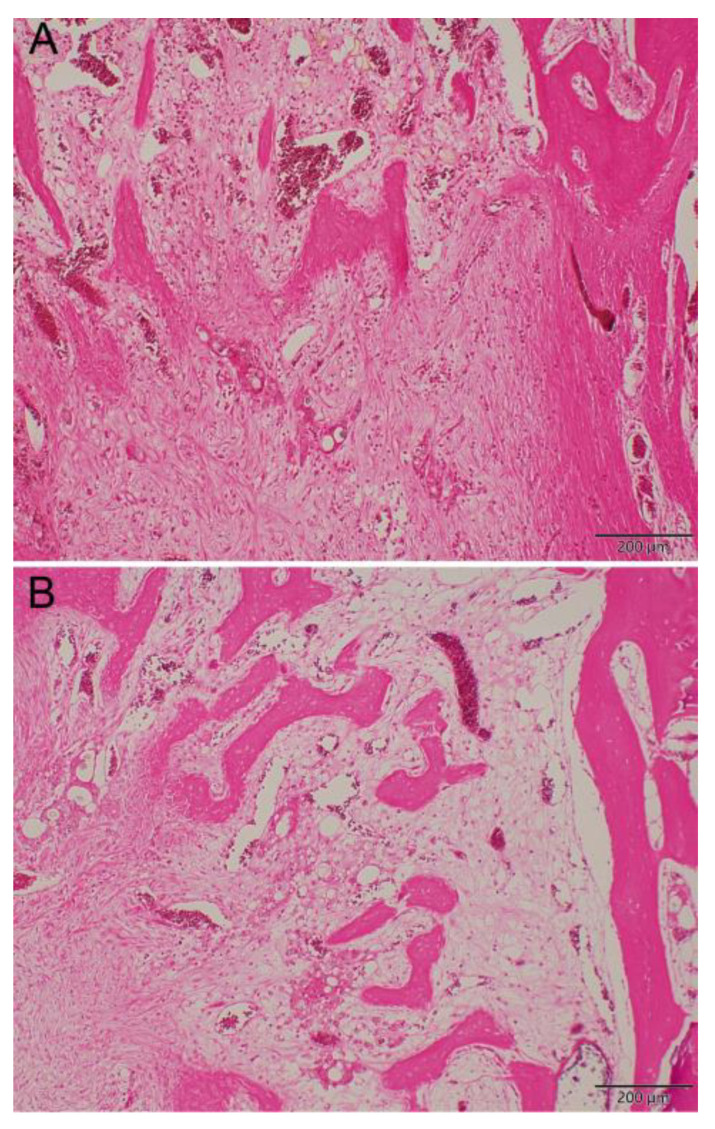
Histologic observation. More mature bone tissue was shown in Li-treated group (**B**) than non-Li-treated group (**A**) in the repaired tissues of the specimen at 8 weeks after bead implantation (hematoxylin and eosin stain; original magnification, ×100).

Figure 10 shows the repaired tissues of the sample at 8 weeks after implantation. Newly formed bone was observed in the control group (Figure 10A, 100×, H&E stain) and the LiCl-bead-treated group (Figure 10B, 100×, H&E stain). More mature bone tissues were observed in the Li-bead-treated group (Figure 10B) than in the non-Li-bead-treated group (Figure 10A).

## 3. Discussion

PLGA is one of the most suitable biodegradable polymer materials for the synthesis of tissue engineering drug delivery devices [18,19,20]. The material is biodegradable and biocompatible, has a large range of erosion times, and is mechanically adjustable. Drug release kinetics can be controlled by the polymerization rates of lactide and glycolide as well as the molecular weight of PLGA [11,21]. The degradation of PLGA results in lactic acid and glycolic acid, which are ultimately degraded to CO_2_ and H_2_O [22]. PLGA is an FDA-approved polymer that is widely used for the control of small molecule drugs, proteins, and other large molecules. Therefore, we selected PLGA as the carrier material for vancomycin-containing beads.

Bacterial infections during surgery can be destructive and are often associated with considerable morbidity and poor functional outcomes. Treatment of postoperative infection requires surgical debridement, removal of implants and all necrotic tissue, and the administration of systemic antibiotics. The delivery of local antibiotics via the use of antibiotic-impregnated biodegradable beads has been proposed to provide the sustained release of antibiotics to infected areas, replacing intravenous antibiotic infusion. In this study, we developed PLGA-LiCl-vancomycin beads for the treatment of bone infection. PLGA has been demonstrated to be an excellent material for various healthcare applications, including tissue engineering, regenerative medicine, the fabrication of cardiovascular stents, and orthopedic interventions [11,12,22,23,24], due largely to its favorable biocompatibility and safe degradation products [23]. PLGA is a degradable polymeric material with superior properties that has been extensively researched. The material has a glass transition temperature of 40–60 °C and a melting temperature of 262 °C, which is suitable for our fabrication process with a hot compression mold at 55 °C (Figure 1). Furthermore, because of the absence of organic solvents during bead preparation, these vancomycin/PLGA beads reduce the problems caused by organic solvents, which destroy drugs or residues in the body, and are thus applicable for clinical treatment for the treatment of infections in bone tissue.

The release curves of vancomycin from the PLGA-vancomycin-LiCl delivery beads are shown in Figure 2. Gradual release of vancomycin was observed from the biodegradable drug delivery beads over the entire duration, remaining above the breakpoint sensitivity level through Day 42 in vitro, and vancomycin release was most obvious during the first 48 h. The initial burst release of vancomycin was justified by the fast release of vancomycin on the surfaces of the PLGA drug delivery beads [18]. This burst release of vancomycin molecules at the surgical site could successfully eradicate bacteria causing infection, followed by 4 to 6 weeks of constant release above the breakpoint sensitivity level [9,10]. The slower subsequent release could lead to continuous dosing of vancomycin as a long-term therapy. The continuous dosing of drug molecules was attributed to the slow diffusion of vancomycin molecules inside their pores [15,18].

Antibiotic concentrations associated with antibiotic bone cements may cause skeletal cell toxicity and prevent fracture healing [19]. Previous studies have suggested that vancomycin has dose-dependent effects on cells. Local administration of vancomycin at high levels may have cytotoxic effects. However, at lower doses (4–400 μg/mL), vancomycin does not significantly impair osteogenic proliferation or function [24,25]. Local vancomycin concentrations of 1000 μg/mL or less had little or no effect on osteoblast replication, and concentrations of 10,000 μg/mL caused cell death. Vancomycin is less toxic than cefazolin to osteoblasts at higher concentrations and may be a better antibiotic for local administration in the treatment of similarly sensitive bacterial infections [26]. Vancomycin and tobramycin at doses greater than 2000 μg/mL severely decreased chondrocyte proliferation. The balance between the targeted microbicidal effects and host cellular toxicity is critical for skeletal cell survival and function [19]. Regarding clinical indications, the microbicidal effect has often been investigated, but its toxicity to osteoblasts has rarely been examined. In the present study, we developed biodegradable drug delivery beads for the treatment of bacterial infections. The highest vancomycin concentration eluted from the PLGA-vancomycin-LiCl delivery beads (Type IV) was 700.3 μg/mL in vitro (Figure 2), which was lower than the inhibitory concentration of vancomycin on osteoblast replication (1000 μg/mL) [20]. Our data suggest that PLGA-vancomycin-LiCl delivery beads may be a good choice for local administration in the treatment of similarly sensitive bacterial infections.

Bone healing is a complex physiological process that is initiated and controlled by growth factors such as bone morphogenetic protein-2 (BMP-2). Because the sintering temperature for hot compression molding (55 °C) is not suitable for the rhBMP-2 protein, we used three steps of compression sintering and ultrasonic welding techniques to manufacture rhBMP-2 containing polymer capsules in our previous study [11]. Since the rhBMP-2 or Wnt 3a proteins are costly and rapidly lose their activity, in the present study, we further investigated the possibility of replacing them with inexpensive commercially available Wnt agonists, specifically lithium chloride (LiCl), for certain applications of MSCs. Wnt signaling has been shown to promote the osteogenesis of MSCs. LiCl upregulates, Wnt signaling, thus increasing the osteogenic capacity of MSCs [27]. In the present study, we used LiCl as an osteogenic enhancer of MSCs and used compression sintering techniques to manufacture LiCl containing polymer beads. The release curves of Li from the PLGA-vancomycin-LiCl delivery beads are shown in Figure 3. Gradual release of Li was observed from the Type II beads for 10 days, Type III beads for 14 days, and Type IV beads for 28 days. Lithium release was the most obvious during the first 48 h. The melting points of LiCl and vancomycin were 614 °C and 175 °C, respectively. The sintering temperature was set at 55 °C, which was higher than the glass transition temperature of the polymer (40–60 °C), but low enough to avoid destroying vancomycin and LiCl.

*Staphylococcus aureus* (*S. aureus*) is the most widespread bacterial etiology identified in traumatic and iatrogenic infections [12,28]. We measured the breakpoint sensitivity of vancomycin in the collected fluid against *S. aureus* (ATCC 259523). Figure 4 shows that the sample inhibition zone and relative activity were greater for Type IV beads than Type I beads for D1, D4, and D7, which suggests that the bactericidal effect of vancomycin was enhanced by Li cotreatment in Type IV beads compared with Type I beads. Vancomycin is a type of glycopeptide antibiotic. The main bactericidal function of vancomycin is to inhibit bacterial cell wall synthesis and disturb the osmotic ability of the cell membrane in *S. aureus* [26]. The permeability of the bacterial cell membrane and the synthesis of RNA can be modified [29,30]. LiCl increases the solubilization of the cell wall structure [31], so it may increase the bactericidal effect of vancomycin.

After treatment of MSCs by collected eluant from PLGA-vancomycin-LiCl beads, the dose-dependent release of Li promoted the osteogenesis of MSCs by increasing osteopontin (OPN) (Figure 5A) and Runx2 mRNA (Figure 5B) expression. In addition, the released Li was observed to promote the osteogenesis of MSCs by increasing the phosphorylation of the GSK-3β protein (Figure 6). LiCl inhibits GSK3β activity and thereby stabilizes free cytosolic β-catenin, thus leading to the intracellular accumulation of β-catenin. β-catenin subsequently translocates into the nucleus to promote the osteogenesis of MSCs by upregulating Runx2 protein expression [32,33]. We observed that released Li stimulated MSC differentiation (Figure 6), and previous studies have shown that Li is not carcinogenic [26] or mutagenic [31]. These attributes make Li a potential supplement for MSC in vitro culture medium optimization.

Although the use of antibiotic-releasing bone matrices [8,9,10,19] and lithium salts [15,17] in bone regeneration is well documented in the literature, the simultaneous effects of vancomycin and Li on bone regeneration have rarely been reported. In the present study, gradual release of vancomycin was observed from the biodegradable drug delivery beads over the entire duration, remaining above the breakpoint sensitivity level through Day 42, and vancomycin release was most obvious during the first 48 h (Figure 2). This burst release of vancomycin molecules at the surgical site could successfully eradicate bacteria causing infection. The slower subsequent release could lead to continuous dosing of vancomycin as a long-term therapy. The continuous dosing of drug molecules was likely due to the slow diffusion of vancomycin molecules held inside their pores [15,18]. The bactericidal effect of vancomycin was enhanced by Li cotreatment in Type IV beads compared with Type I beads (Figure 3 and Figure 4, Table 1). After treatment of the MSCs by collected eluant from the PLGA-vancomycin-LiCl beads, the released Li was shown to promote the osteogenesis of the MSCs by increasing the mRNA expression of osteopontin (OPN) (Figure 5A) and Runx2 (Figure 5B). In addition, the released Li was shown to increase the phosphorylation of the GSK-3β protein and upregulate the expression of the Runx 2 protein (Figure 6). Our results showed the simultaneous action of vancomycin and Li in bone regeneration (Figure 4, Figure 5 and Figure 6). These two elements indicate that increased Li tends to promote the ossification of MSCs; however, additional observations are still needed to demonstrate this effect.

Lithium significantly enhances bone formation in rats [34] and accelerates fracture healing clinically [35]. In addition, Li is taken up by a variety of tissues, with bone and muscle containing the highest concentrations, making Li especially suitable for treating bone disorders [16]. To investigate the effects of released Li and vancomycin in vivo, we created a rabbit model. A cylindrical cavity was made at the distal end of the right femur, and one composite delivery bead was inserted into the cavity (Figure 7). After implantation of the PLGA-vancomycin-LiCl beads for 7, 14, 28, and 42 days, the fluid content was aspirated from each femoral cavity, and the concentrations of Li (Figure 8) and vancomycin (Figure 9) were quantified.

Figure 8 shows the Li concentrations measured in the bone cavity tissue. Gradual release of Li^+^ ions from Type IV beads was observed for 42 days in vivo. Because Li molecules can be used as Wnt signaling activators to promote the osteogenesis of MSCs, more mature bone tissues were observed in the Li-bead-treated group (Figure 10B) than in the non-Li-bead treated group (Figure 10A). The previously mentioned delivery systems for vancomycin are very useful for bone reconstructive surgery [5,12,19]. The localized release of a powerful antibiotic such as vancomycin during the early period following surgery (1–6 days) can inhibit bacterial infection, thus preventing severe complications and implant failure. However, for acute cases such as osteomyelitis, the drug delivery systems should be able to deliver therapeutic doses of antibiotics for at least 2 weeks. In the present study, long-term medication was provided by PLGA-Li-vancomycin beads. The beads were able to deliver therapeutic doses of vancomycin for up to 6 weeks in vivo (Figure 9), thus controlling the bone infection.

In the present study, we offer a convenient method for multiple deliveries of the Wnt agonist Li and antibiotics via biodegradable PLGA-Li-vancomycin beads to meet the specific antibiotic requirements for patients with osteomyelitis. Although the current study has generated promising preliminary data, some limitations should be noted. For example, we used a noninfected animal model; therefore, it is unclear whether the PLGA-Li-vancomycin beads might perform differently in infected tissue. Further evaluation of PLGA-Li-vancomycin beads in an animal model of *S. aureus* infection is necessary to address this limitation.

## 4. Materials and Methods

The experimental protocol was approved by the Institutional Animal Care and Use Committee of the Chang Gung Memorial Hospital (No: 2017120803, the approval date of our animal experiments was 20180528). The PLGA polymer Resomer RG503 (lactide:glycolide, 50:50) (molecular weight: 33,000 Da) was purchased from Boehringer Ingelheim (Ingelheim am Rhein, Germany). Powdered vancomycin and lithium chloride (LiCl) were purchased from Sigma. A Millipore Ultrapure Water System (Watford, UK) was used to obtain HPLC-grade water. Acetonitrile was purchased from Mallinckrodt Baker. Heptane sulfonic acid was purchased from Fisher Ltd. Commercially available human mesenchymal stem cells (MSCs) cell line and MesenPRO RS™ medium were purchased from Invitrogen.

### 4.1. Fabrication of a Biodegradable PLGA Drug Delivery System for Antibiotic and Lithium Delivery

To ensure homogeneity, PLGA (125 mg), vancomycin (25 mg), and LiCl (10, 50, or 100 mg) were first blended using a dry mixer for 20 min. The mixtures were then loaded into molds and hot compression molded at 55 °C (Figure 1). Each of the antibiotic beads was incubated in 5 mL of PBS (pH 7.4) at 37 °C for 24 h to assay the elution rate of vancomycin and lithium from PBS in vitro. At each 24 h interval, the PBS was drawn, and the beads and were re-submerged in fresh buffer. The removed PBS with vancomycin and lithium was frozen at −70 °C until study.

### 4.2. In Vitro Analysis of Vancomycin and Lithium Release

The concentrations of vancomycin in the collected fluid were determined by a high-performance liquid chromatography (HPLC) assay method. The separating column was a C8 Symmetry HPLC column (Waters Asia Ltd., Singapore). The mobile phase contained 0.01 M heptane sulfonic acid (Fisher, Loughborough, UK) and acetonitrile (Mallinckrodt Baker, KY, USA; 85/15, *v*/*v*). Absorbance was monitored at 280 nm under a flow rate of 1.4 mL/min.

The concentrations of lithium in the collected fluid was determined by the Dimension^®^ EXL™ 200 Integrated Chemistry System (www.siemens.com/diagnostics, accessed in 31 October 2017) in Division of Laboratory Medicine, Chang Gung Memorial Hospital. Briefly, the Li detection method employs a patented compound, 7-Nitro-2,12-dicarboxyl-16, 17-dihydro-5H,15H-dibenzo[b,i] [1,4,5,7,8,11] dioxatetraazacyclotetradecine (lithium dye) that reacts with the Li^+^ ion in an alkaline mixture of water and dimethylsulfoxide to form a non-covalent binary complex, Lithium Dye-Li Complex (absorbs at 540 nm). The change in absorbance at 540 nm due to the formation of this non-covalent binary complex is directly proportional to the lithium concentration in the sample and is measured using a bichromatic (540 and 700 nm) endpoint technique.

### 4.3. In Vitro Analysis of Antibacterial Activity in the Collected PBS with Vancomycin and Lithium

*Staphylococcus aureus* (*S. aureus*) (ATCC 259523) was cultured in static at 37 °C for 24 h in 12 mL of broth (LB broth, GIBCO, Life Technologies Corporation, Grand Island, NY, USA). Minimal inhibitory concentration (MIC) of vancomycin in the collected fluid against *S. aureus* was measured with an antibiotic tube dilution method in Cation-Supplemented Mueller–Hinton Broth (Difco Laboratories, Detroit, MI, USA). Medium was diluted serially twice in tubes containing 0.5 mL broth. The *S. aureus* inocula for each series of tubes was 0.5 mL of an overnight culture containing 5 × 10^5^ colony-forming units/mL. MIC was defined as the lowest antibiotic concentration preventing turbidity after 24 h of incubation at 37 °C.

Relative activity of vancomycin with or without Li against *S. aureus* was determined by an antibiotic disk diffusion method applied to the nutrient broth. Each sample was diluted or concentrated to 50 mg/mL. Solution (8 mL) from each buffer sample was harvested daily and pipetted onto 7 mm disks, which were placed on the nutrient agar plates (Difco Laboratories) seeded with a lawn of *S. aureus*. Zones of inhibition were measured with a micrometer after 16 h to 18 h of incubation at 35 °C. The equation for relative activity was relative activity (%) = (diameter of sample inhibition zone − disk diameter)/(diameter of maximum inhibition zone − disk diameter).

### 4.4. Effects of the Collected PBS with Vancomycin and Lithium on Osteogenesis of MSCs

StemPro^®^ BM MSCs were purchased from Gibco (Life Technologies Corporation). Primary MSCs were expanded with 2% FBS MesenPRO RS™ medium (Gibco) containing 2 mM L-glutamine and 5 μg/mL gentamicin in T-75 flasks and maintained in an incubator at 37 °C /5% CO2. The media was changed every three days, and the cells were split at 80–90% confluence. The cells were used at early passage (<5 passages) for all experiments.

Approximately 2.5 × 10^5^ MSCs were seeded onto a 100 mm cell culture dish. Daily, 100 μL of the collected liquid with or without vancomycin and lithium was transferred to the medium. After culturing for 14 d, the cultured cells were rinsed with PBS. Total RNA was extracted using a Qiagen RT kit (Qiagen, Germantown, MD, USA). To detect the Runx 2, osteopontin (OPN), osteocalcin (OCN), and GAPDH RNA transcripts, cDNA was analyzed using an ABI PRISM 7900 sequence detection system and TaqMan PCR Master Mix (Applied Biosystems, Foster City, CA, USA). Total protein was extracted using M-PER protein extraction reagent (Thermo, Waltham, MA, USA). *Protein* sample quantitation with a protein assay kit (Pierce Biotechnology, Rockford, IL, USA), separated with 7.5% SDS-PAGE, and protein expression were detected for phosphor-GSK-3β (Ser9) (Abcam, Cambridge, UK), GSK-3β (Cell Signaling, Danvers, MA, USA), and Runx 2 (Millipore, Burlington, MA, USA).

### 4.5. Fabrication of a Biodegradable PLGA Drug Delivery System for Antibiotic and Lithium Delivery

PLGA (125 mg), vancomycin (25 mg, Sigma, Livonia, MI, USA), and LiCl (100 mg, Sigma) were loaded into molds, then with hot compression, were molded at 55 °C to form biodegradable vancomycin and Li delivery systems for in vivo study.

### 4.6. Animal Model of Bone Defect

Eight adult male New Zealand white rabbits weighing 2.9–3.5 kg were used. All animal procedures were reviewed and approved by the Institutional Animal Care and Use Committee of the Chang Gung Memorial Hospital (No: 2017120803). The methods were carried out in accordance with the approved guidelines. At first surgery, a cylindrical cavity (15 mm × 12 mm × 10 mm) was made at the side of the right femur distal end and obliterated with a poly (methyl-methacrylate) (PMMA) spacer. The wound was closed with 3-0 nylon sutures. After 2 weeks, the PMMA spacer was removed, and one PLGA drug delivery bead was inserted into the cavity, and then the wound was closed. After the implantation of the PLGA drug delivery system, the fluid content was aspirated from each femoral cavity on days 7, 14, 28, and 42 after implantation. Four rabbits in each group were killed at 8 weeks for histologic observation.

#### 4.6.1. Li Detection

The concentrations of lithium in the collected fluid was determined by the Dimension^®^ EXL™ 200 Integrated Chemistry System in Chang Gung Memorial Hospital, as previously described.

#### 4.6.2. Vancomycin Detection by HPLC

The concentrations of vancomycin in the collected fluid were determined by a HPLC assay method, as previously described.

### 4.7. Immunohistochemical Evaluation

After decalcification, the specimens harvested from femoral cavities were embedded in paraffin, cut into 5-μm thick sections, and processed for H&E staining.

### 4.8. Statistical Analysis

Student’s t-test was used to analyze the LiCl-treated group/non-LiCl-treated group ratio in this study. Data were represented as mean ± standard division (SD). The p-values for the Student’s *t*-test were performed using the SPSS software package (Version 12.0, SPSS Inc., Chicago, IL, USA). A *p*-value of <0.05 was considered statistically significant.

## 5. Conclusions

Because Li^+^ ions can be used as a WNT signaling activator to promote the osteogenesis of MSCs, more mature bone tissues were observed in the Li-bead-treated group than in the non-Li-bead-treated group. This study offers a convenient method for multiple deliveries of WNT agonists and antibiotics by biodegradable PLGA-Li-vancomycin beads to meet the specific antibiotic requirements for patients with bone infections.

## Figures and Tables

**Table 1:** The sample inhibition zone and relative activity in vitro.

Inhibition Zone (mm)			Relative Activity (%)		
Day	Type I Beads	Type IV Beads	Day	Type I Beads	Type IV Beads
1	13	15	1	100%	100%
4	10	12	4	50%	62.5%
7	8.5	10	7	25%	37.5%
14	8	9	14	16.7%	25.0%
21	7.5	8.5	21	8.3%	18.8%
28	7.5	8	28	8.3%	12.5%

## Data Availability

Data is contained within the article.
